# Editorial: Methods in cognitive neuroscience: dance movement 2023

**DOI:** 10.3389/fnhum.2024.1542595

**Published:** 2025-01-09

**Authors:** Andrea Orlandi, Kohinoor M. Darda, Beatriz Calvo-Merino, Emily S. Cross

**Affiliations:** ^1^Department of Psychology, Sapienza University, Rome, Italy; ^2^IRCCS Santa Lucia Foundation, Rome, Italy; ^3^School of Psychological Sciences, Macquarie University, Sydney, NSW, Australia; ^4^Advancement and Research in the Sciences and Arts (ARISA) Foundation, Pune, MH, India; ^5^Cognitive Neuroscience Research Unit, Department of Psychology, City, University of London, London, United Kingdom; ^6^Professorship for Social Brain Sciences, Department of Humanities, Social and Political Sciences, ETH Zürich, Zürich, Switzerland

**Keywords:** dance neuroscience, research methods, protocols, social cognition, non-human agents, movement kinematics, dyadic interactions, functional connectivity

Over the past few decades, dance has emerged as a unique medium to explore the intricacies of the human brain, mind, and body. Dance provides a dynamic lens to examine neural representations of complex movement, and understand individual, cultural, and universal factors influencing our emotional and aesthetic evaluation of movement. Dance research has vast potential and uses a wide range of neuroscientific methods—including psychological and brain measures, behavioral training procedures, and kinematic analyses. Yet the field is still in its infancy, grappling with the lack of standardized methods and guidelines that can ensure accessibility and scientific rigor. This absence of methodological standardization and frameworks can threaten the growth of this promising domain, limiting its ability to address the complexity of studying human movement in an artistic context. Recent efforts aim to focus on scientific rigor, embrace cross-cultural perspectives beyond *western* dance and culture, and expand understanding of two-body and multiple-body aesthetics, paving the way for a more holistic understanding of human movement and cognition in dance.

In response to these challenges, this Research Topic, part of the Methods in Frontiers in Human Neuroscience series, highlights novel methodological advancements applicable to the investigation of the cognitive neuroscience of dance and complex movement. It focuses on neuroaesthetics and the social, affective, and cognitive dimensions of dance. A key aim is to foster interdisciplinary dialogue among experts from various fields, drawing on strengths from research in empirical aesthetics, cognitive neuroscience, social cognition, and computer vision. The five studies showcased in this Research Topic embody this interdisciplinary approach, incorporating diverse techniques such as neuroimaging and computational kinematic analysis, and implementing comprehensive research protocols.

Baker et al. introduce a computational approach for classifying Hip Hop dance genres, using 17 full-body movement features derived from 3D joint positions. Their findings highlight the significance of features like body expandedness and sharp movements frequency in distinguishing between genres. This method outperformed simpler machine learning classifiers and revealed both convergences and divergences when compared with human participant performance, suggesting promising potential for future applications in other movement domains. Additionally, the authors employed Latent Semantic Analysis to reduce the feature dimensionality and characterize genres through key aspects like vertical movement (bounce), momentum, and rhythmic regularity, providing new insights into Hip Hop dance evolution.

Moffat et al. use kinematic analysis together with questionnaires to investigate the relationship between dyadic-level embodiment (body competence and body perception scores) and movement features (synchrony and complexity) during a movement mirroring game. Participants move their arms spontaneously, while a confederate closely matched their arm movements in space and time, with the roles then reversed. Results revealed that when the more experienced member of the dyad (the confederate) followed the participant's movements, the dyad achieved greater synchrony. Notably, synchrony—but not complexity—was positively associated with dyadic body competence scores. This study highlights the importance of considering dyadic-level embodiment to gain insight into behaviors in interactive social contexts, which may extend to interactions with non-human agents as well.

Directly addressing the theme of non-human agents and dance, Darda et al., explore how stimulus and knowledge cues related to human animacy affect the aesthetic appreciation of dance. Participants watched Bharatanatyam dance videos performed by human and robotic avatars, with variations in whether the choreographies were human- or computer-generated (stimulus cues). The authors also manipulated participants' beliefs about whether the movement and choreography were human- or computer-generated (knowledge cues). The study found that participants preferred choreographies they *believed* to be human-generated, but favored robotic performances when both movement and choreography were believed to be computer-based, suggesting a preference for agent congruence. Factors such as dance and technology expertise, along with attitudes toward AI, also influenced participants' aesthetic judgments, providing insights into how technological interventions impact dance perception.

The next study we highlight was by Yang et al., who use functional neuroimaging (fMRI) to conduct a resting-state functional connectivity analysis to examine how long-term dance training (at least 10 years at Art Universities in Taiwan) influences the relationship between brain networks associated with metacognition, brain networks linked to dance observation, and general creativity indices. The results showed that dancers exhibited increased functional connectivity between metacognitive networks and subcortical regions associated with dance observation, including the putamen, globus pallidus, anterior insula, and posterior cerebellum. The strength of this connectivity was modulated by creativity indices of originality and flexibility, possibly supporting better integration and coordination of creative cognitive processes in dancers.

Finally, Poikonen et al. present a study protocol for a randomized controlled trial examining the effects of a 12-week mixed physical exercise program incorporating creative movement, called “InMotion”, for adults with schizophrenia. This intervention, set to be delivered alongside standard pharmacological treatment, emphasizes physical exercise, enjoyment, social interaction, and playful creativity in a safe setting. Outcomes will be measured using questionnaires, behavioral, physiological, and neural assessments at baseline, mid-treatment, post-treatment, and during two long-term follow-ups. The intervention aims to reduce negative schizophrenia symptoms (such as blunted affect and emotional withdrawal) compared to a control group and is further anticipated to modulate physiological and neural markers, enhance physical health and function, reduce sedentary behavior, improve cognitive control and emotion regulation, and increase mobility while decreasing movement rigidity.

Taken together, these papers reflect the strong potential of dance as both a research subject and a tool for understanding human cognition. Findings on human animacy and neuroplastic changes due to dance that impact creativity cognition underscore the importance of dance to investigate not only human movement, but also fundamental aspects of human cognition. Intervention studies point to a future where dance remains not just a performance art form or research tool, but also a tangible medium for promoting mental health and wellbeing. Robust evidence already shows how dance bridges neuroscience, psychology, performing arts, social cognition, aesthetics, and applied interventions. Looking ahead, the integration of diverse and rigorous methods, cross-cultural and interdisciplinary perspectives, alongside technological advancements and further collaboration between artists and scientists, promises to propel dance neuroscience into exciting new territories where the innerworkings of the human brain, mind, and body are further illuminated.

